# Forecasting water quality parameters using artificial neural network for irrigation purposes

**DOI:** 10.1038/s41598-021-04062-5

**Published:** 2021-12-24

**Authors:** J. I. Ubah, L. C. Orakwe, K. N. Ogbu, J. I. Awu, I. E. Ahaneku, E. C. Chukwuma

**Affiliations:** 1 Department of Agricultural and Bioresources Engineering, Nnamdi Azikiwe University, Awka, Nigeria; 2 National Centre for Agricultural Mechanization, Ilorin, Nigeria; 3 Michael Okpara University of Agriculture, Umudike, Nigeria

**Keywords:** Ecology, Environmental sciences, Hydrology

## Abstract

This study was aimed at analyzing the water quality of Ele River Nnewi, Anambra State for irrigation purposes with a view to predicting a one-year water quality index using Artificial Neural Network (ANN). Water pollution has posed a major problem and identifying the points of pollution in the River system is a very difficult task. To overcome this task, the need to determine the pollution level arose by modeling and predicting four water quality parameters at four (4) different locations using the Artificial Neural Network. These parameters include the pH, Total Dissolved Solids (TDS), Electrical Conductivity (EC), and Sodium (Na), respectively. The water quality results showed that the pH values which ranges from 6.01 to 6.87 were within the FAO standard in all the points for both rainy and dry seasons, whereas the TDS (mg/l), EC (dS/m) and Na (mg/l) parametric values range from 2001 to 2506, 3.01 to 5.76, and 40.42 to 73.45 respectively, were above the FAO standard from point 1 to point 3 and falls within the FAO standard at point 4 with values ranging from 1003 to 1994, 2.01 to 2.78 and 31.24 to 39.44, respectively. However, during the dry season, the TDS, EC, and Na values range from 2002 to 2742, 3.04 to 5.82 and 40.14 to 88.45 respectively, were all above the FAO standard. Generally, the artificial neural network modeled the actual water quality data set very well with good prediction. The training model performance evaluation shows that the R^2^ values ranges from 0.981 to 0.990, 0.981 to 0.988, 0.981 to 0.989 and 0981 to 0.989, for pH, TDS, EC, and Na. The testing model performance shows that the R^2^ value ranges from 0.952 to 0.967, 0.953 to 0.970, 0.951 to 0.967and 0.953 to 0.968, for pH, TDS, EC and Na while the forecast performance evaluation shows that the R^2^ values ranges from 0.945 to 0.968, 0.946 to 0.968, 0.944 to 0.967 and 0.949 to 0.965 for pH, TDS, EC and Na respectively. It was also observed that the Root Mean Squared Error (RMSE) ranges from 0.022 to 0.088, 0.012 to 0.087, 0.015 to 0.085 and 0.014 to 0.084 for pH, TDS, EC and Na, respectively. Information from this study will serve as a guide to researchers on the water quality index for irrigation purposes. Also, it will guide the government and agencies on policy, management and decision-making on water resources.

## Introduction

Water is a widely distributed natural resource and as well, a notable solvent. It supports life and if not properly managed, harbors disease-causing germs and chemical contaminants^4^.Water is very beneficial considering its various uses which socially and economically promote and enhance societal wellbeing^14^.Water supply for domestic and industrial use, leisure and marine life, and agricultural production are all forms in which water can be used. Unregulated waste disposal, soil geological composition, anthropogenic and seasonal shifts all interfere with agricultural production's use of various water sources. The perceived effects of unregulated waste disposal into water bodies used as portable water sources have sparked various studies on effluent discharge^4^. Globally, laws have been established for guidelines to protect water quality for downstream uses. These can be seen in the Food and Agriculture Organization (FAO) water guidelines for irrigation and Standard organization of Nigeria standards for irrigation and consumption purposes. On this note, river basin authorities and state water corporations were established to control water use and provide potable water for beneficial purposes^15^.

Domestic, municipal and agricultural wastes all have an effect on the daily life of water sources in various ways and the impact when strong enough to make water unfit for human consumption or agricultural purposes, then the water is said to be polluted or contaminated^12^. Pollution of a water body is defined as the influx of contaminated substances or an increase in the concentrations of normal water components to levels that disrupt the water body's delicate eco-equilibrium and impair its natural cleaning capacity^4^. Water pollution has harmful effects on living organisms especially Man due to its various diseases. It has devastated the aquatic ecosystem so much which calls for possible remedies. Life in an aquatic environment is said to be balanced when these biotic and abiotic elements are in ideal conditions, and the water body is colorless, odourless, and tasteless. Pollution alters these factors of a stable eco-system, making the water unfit for human, industrial, and agricultural uses^16^. The characteristics of surface and ground water drawn from community sources are complex. Despite the fact that few rivers are suitable for public use without treatment, some are not. To get water quality up to the level needed for public usage, the majority of water supplies must be properly managed^14^.Water quality varies depending on the source of contaminants, the time of year (season), and the river's geological formation. During the rainy season, increased rainfall causes constant dilution of groundwater movement, making the river's physico-chemical parameters almost as good as those of fresh water. Human faeces, food remnants, and a mixture of organic and inorganic solid wastes and salts are all contaminants in river waters^4^. Depending on the source of the river and its tributaries, a river is a significant receptacle for all effluent discharges from domestic activities.

However, Ele River located at Nnewi, Anambra State has been adversely polluted by effluent discharges from industries located at the upland of the River. Many factories are situated near water bodies, presumably to make the discharge of effluents and other wastes into them as simple as possible^19^. The water quality of this River is being affected by this pollution thereby affecting its use for irrigating nearby farmlands. To determine the pollution level of the River so as to check is suitability for farm irrigation and at the best point to obtain a good water supply, the water quality parameters of the River were modeled and forecasted using an Artificial Neural Network (ANN). The water quality parameters selected for analyses were based on the problems most soil commonly encountered and these are related to salinity, water infiltration rate, ion toxicity and a group of other miscellaneous problems. For salinity, Total Dissolved Solid (TDS) and Electrical Conductivity were selected; for Toxicity, Sodium was selected and for miscellaneous problems, pH was selected. Therefore, a total of four (4) parameters at four (4) different points along the river course were examined and predicted in this study. Thus, the objective of this study is to analyze the water quality of Ele River Nnewi, Anambra State for irrigation purposes by predicting the water quality index using Artificial Neural Network (ANN) for 12 months. Four water quality parameters were considered at four (4) different sampling points.

Salinity problems deal with the **s**alts in the soil or water which reduces water availability for crops to such an extent that yield is affected. The ion toxicity problems in the water accumulate in a sensitive crop to concentrations high enough to cause crop damage and reduce yields. The miscellaneous problems when in the water cause excessive nutrients which reduce yield or quality; unsightly deposits on fruit or foliage reduce marketability; excessive corrosion of equipment increases maintenance and repairs.

Artificial neural network is a technique with a flexible mathematical structure that is capable of identifying complex non-linear relationships between input and output data when compared with other classical modeling techniques^9^. It comprises methods for analyzing time-series data so as to extract characteristics of data and forecast future events based on known past. Water quality index of Paraka Lake, India was predicted using an artificial neural network^14^.

ANN is composed of a large number of simple processing units, each interacting with others via excitatory or inhibitory connections. The ANN technique is flexible enough to accommodate additional constraints that may arise during its application. Moreover, the ANN model can reveal hidden relationships in historical data, thus facilitating the prediction and forecasting of water quality^8^. In ANN modeling, different layers in the program can be distinguished as; the input layer—connecting the input information to the network, the hidden layer- acting as the intermediate computational layer between the input and output layer and the Output layer—producing the desired output which is in this case the Water Quality Index (WQI) as shown in Fig. 3.

A feed-forward multilayer perceptron is the ANN architecture that was employed in this study. This structure of an artificial neural network has been proven to be the best neural network structure for hydrological modeling^18^.

The objective of this study was to analyzing the water quality parameters of Ele Rivers at four (4) different points of 20 m apart with a view to predicting the next one-year water quality parameters using Artificial Neural Network (ANN) for irrigation purposes.

Kargar et al.^8^, predicted the longitudinal dispersion coefficient in natural streams using empirical models and machine learning algorithm. Though different ANN shields have been explored for hydrological and agricultural prediction but little is known on agricultural research work using Alyuda ANN shield other than the hydrological application. Therefore, the application of Alyuda ANN shield for irrigation prediction applications in this study make this study unique as it tends to reveal its capabilities for agricultural uses.

Recently in Nigeria, due to increasing economic crisis since 2014 that has led to the crash in the price of oil in the global market culminating in an economic recession since the first quarter of 2016 has once again raised the clarion call for the diversification of the Nigerian economy from its near-absolute dependence on the oil industry to agriculture. Hence, agriculture irrigation accounts for 70% of water use worldwide and over 40% in many Organizations for Economic Co-operation and Development countries^11^. However, in recent years, agricultural regions around the globe have been subject to extensive and increasing water constraints due to global climatic change. This and many more brought about an accelerated need for alternative sources of river water for irrigation purposes. Therefore, the evaluation of water quality parameters and their forecast becomes very important. Information from this study will serve as a guide to researchers on the water quality index for irrigation purposes. Also, it will guide the government and agencies on enacting policies and decision-making on water resources and its management.

## Materials and methods

### Study area

**Figure 1:**
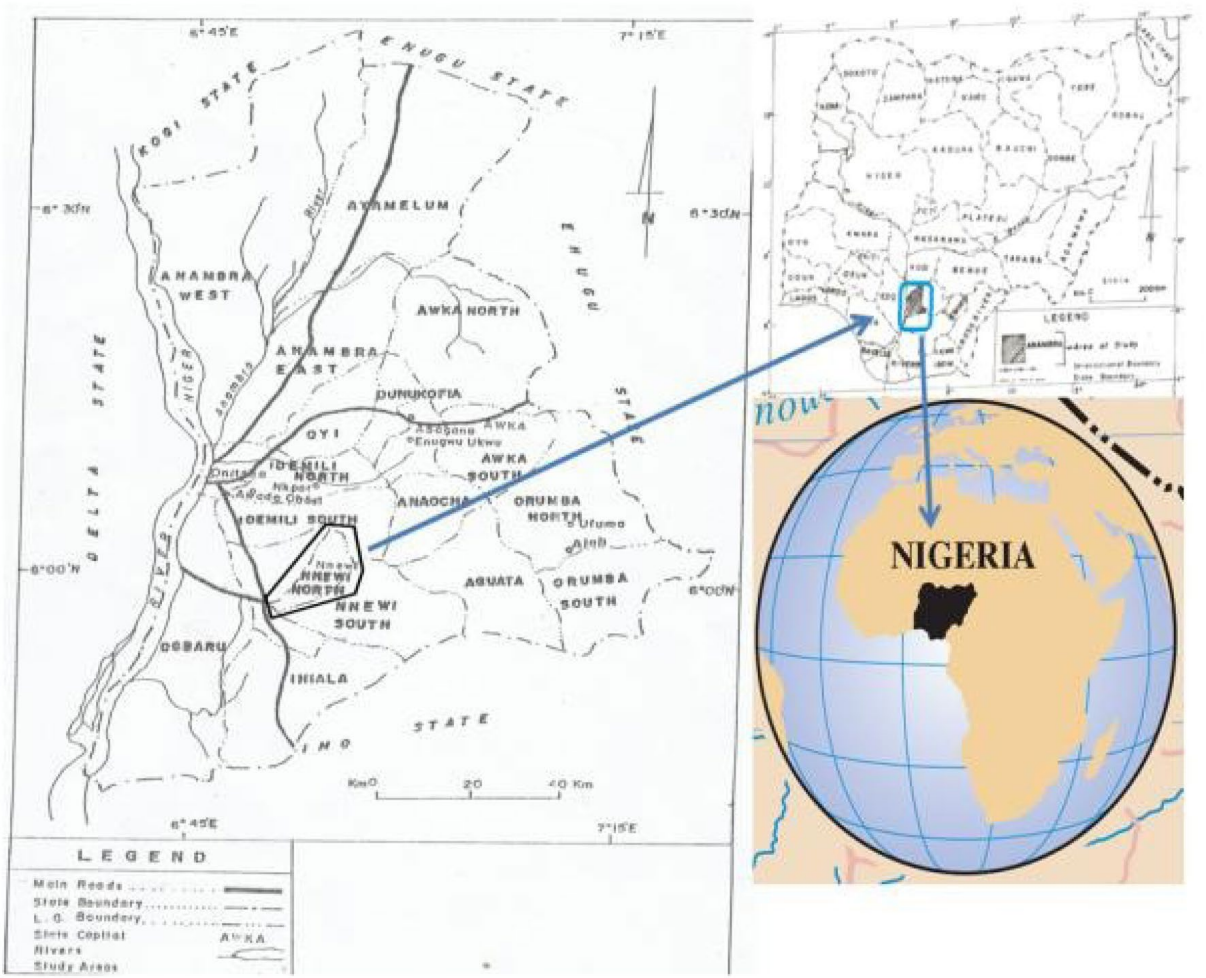
Map of Anambra showing Nnewi. Source: Chukwuma et al.^13^.

The study area is Ele River Nnewi, Anambra State. It lies on Longitudes 6° 91' E and 6° 55' E and Latitudes 6° 16' N and 6° 55' N. It has an average annual rainfall of 200 mm and mean temperature of 27 °C (Fig. 1). The months of April to October experience heavy rainfall, while low rainfall, higher temperature and low humidity characterize the months of November to February. Ele River is important to surface water since it serves various purposes including agricultural and domestic needs for the residents.

**Figure 2:**
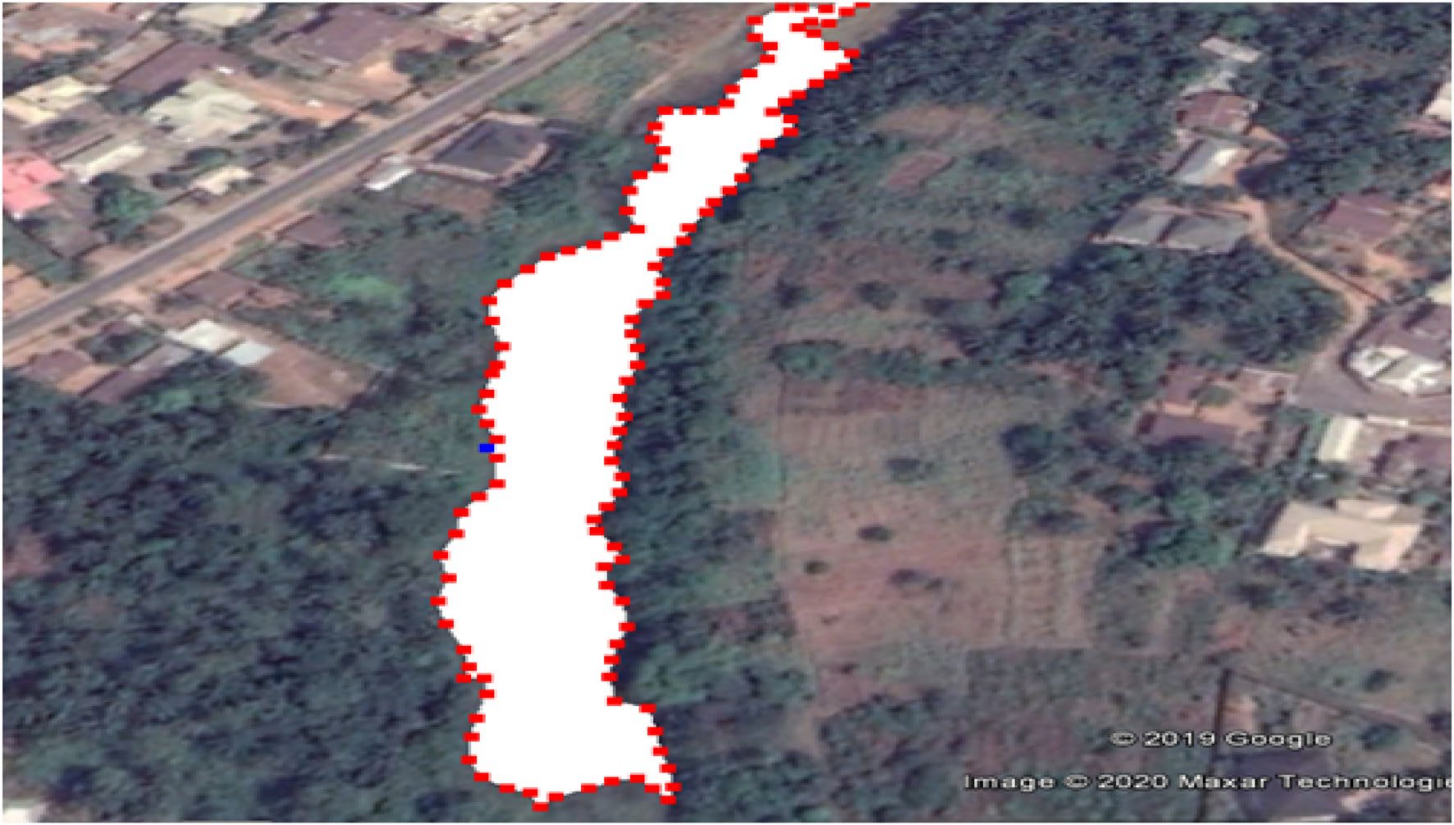
Visualized raster image showing Ele River and the industrial zone and major tributary. Source: GIS Google image.

**Figure 3:**
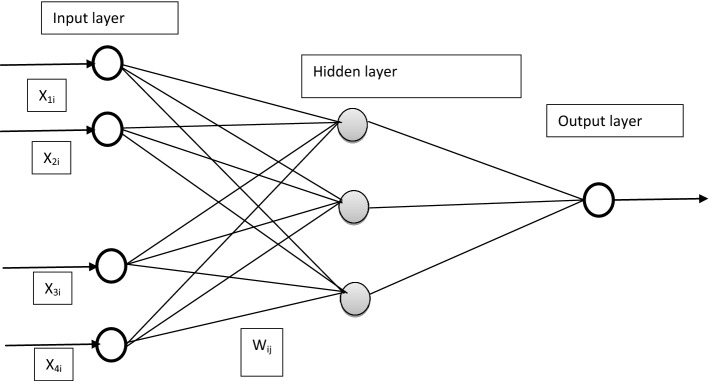
Schematic diagram of feed forward multilayer neural network architecture (x-input layers; w-synaptic weights).

The city spans over 1076.9 square miles (2789 km^2^) in Nnewi, Anambra State. The Ele River is located very close to Industrial Clusters at Umudim Nnewi. Notable among them are, Chicason group of companies, Kotec industries Limited, Innoson Vehicle Manufacturing etc. Effluents from these industries are discharged into Ele River (Fig. 2, 3).

### Point sampling and analysis

Ele River based on the preliminary survey was divided into four (4) sampling points putting into consideration the point of discharge which is very close to the Chicason Group of Companies discharge outlet**.** These four (4) sampling points were named P1, P2, P3 and P4, so as to determine the pollution level from the point source to a particular distance considering ion dissolution and sediment transportation from discharge point to sampling point 4. P1 and P2 were located upstream while P3 and P4 were located downstream. Distance from each sampling point was 20 m and duplicate water samples were collected using 1.5 L sterile plastic can. Monthly water samples were collected from May 2015 to April 2019 for each point. The water quality parameters considered in this study include the pH, TDS, EC, and Na, respectively. Each parameter test was replicated and an average was taken as the true value for the parameter. The samples collected were properly labeled and transferred to Springboard Scientific Laboratory Awka for preservation and analysis. The physical parameters such as the pH, TDS, and EC were determined in-situ. The methods employed in the study are as described below:

#### Direct reading engineering method (Drem)

This was used mainly for the analysis of physical parameters such as pH, total dissolved solids (TDS), and electrical conductivity (EC). The HaccMultimeter (model 150) was used to measure these parameters in situ.

#### Atomic absorption spectrophotometric method

This was used in the determination of the metallic elements such as *Sodium (Na)* according to the methods of Ajali^2^. The results of the analysis were subjected to Artificial Neural Network (ANN) modeling to predicting the next one-year water quality parameters for irrigation.

### Development of artificial neural network

Alyuda artificial neural network shield was adopted in this study based on the architectural and training declaration. A feed-forward multilayer perceptron architecture and supervised feed-forward back-propagation training algorithm were adopted in the development of the neural network. Artificial intelligence (AI)-based models have attracted the interest of numerous researchers over the last two decades due to their capacity to identify complicated and nonlinear correlations among variables^17^**.**

#### Feed-forward multilayer perceptron (FFMLP)

Practically, this network architecture contains a number of layers namely; the input, hidden and output layers, respectively. The architecture determines the number of connection weights and also the way information flows through the network. The determination of the best network architecture is one of the difficult tasks in the artificial neural network model building process but one of the most important steps that must be taken. In this study, the river quality parameters at times t_-n_ was used to predict the river quality parameters at time t_n+1_. The Neuron class describes an entity with an (x, y) location that manages an array list of neurons, as well as its own location that are drawn relative to the network’s center.

A connection object was also created to connect the neurons from one layer to another. The connection object was made to connect neurons from the preceding layer to the succeeding layer. A new function called ‘connect’ was therefore added in the neuron class to connect objects between the specified neurons.

#### The neural network training

Supervised feed-forward back-propagation training algorithm was adopted in this study. The supervised back-propagation training algorithm endeavours to minimize the error between the desired value and the network output value by changing the values of the synaptic weights in the network through calculating the difference between the network output values and the target values and feeding them back to the network.

The back-propagation class created takes signals from the input layer $${x}_{i}$$ and multiplies it by a set of fully-connected synaptic weights $${w}_{ji}$$ connecting the input layer to the hidden layer using the activation function ($${v}_{j})$$ . The computation forms the pre-activation signal for the hidden layer. The pre-activation signal of the hidden layer was transformed using the output function y($${v}_{j}$$) to form the feed-forward activation signals leaving the hidden layer $${x}_{j}$$ to the next neuron in the next layer, this process continued to the output layer. Difference between the network output values to the desired targets $${d}_{k}$$ was calculated using Eq. (1)

1 $${e}_{k}={d}_{k}-{y}_{k}$$

where: e is the output error, $k$ is the output layer notation, ${d}_{k}$ is the desired value, ${y}_{k}$ is the neural network output value.

The network output induced field also known as the activation of the neurons in the output layer was calculated using Eq. (2).

2 $${v}_{k}(n)=\sum_{j=0}^{m}{w}_{kj}\left(n\right){y}_{j}$$

where: ${y}_{j}$ is the output of the layer previous to the output layer, m is the number of neurons in the previous layer.

Therefore, the actual network output was calculated using Eq. 3.

3 $${y}_{k}\left(n\right)=\varnothing \left({v}_{k}\left(n\right)\right)$$

where $\varnothing$ is the partial derivative of the local induced field.

Once, the trained and test model were satisfactory, one to twelve months’ predictions of the selected water quality parameters was made using the developed ANN model.

### Model performance evaluation

Performance evaluation of the trained artificial neural network model was carried out to have an understanding of how good the developed model was. To evaluate the performance of the artificial neural network model, some statistical measurements were considered. These include; the coefficient of multiple determination (R^2^) and the root mean squared error (RMSE) given by Eqs. (4) and (5), respectively^1^.

4 $${R}^{2}=\frac{{\sum }_{i=1}^{n}\left({Q}_{i}-\overline{Q }\right)-{\left({\widehat{Q}}_{i}-{\grave{Q}}\right)}^{2}}{\sqrt{{\sum }_{i=1}^{n}{\left({Q}_{i}-\overline{Q }\right)}^{2}}{\sum }_{i=1}^{n}{{Q}_{i}-{\grave{Q}}^{2}}}$$

5 $$RMSE=\frac{\surd {{\sum }_{i=1}^{n}\left({Q}_{i}-{\widehat{Q}}_{i}\right)}^{2}}{n}$$

where: ${Q}_{i}$ are the *n* modeled flows, ${\widehat{Q}}_{i}$ are the *n* observed flows, $\overline{Q }$ is the mean of the observed flows and ${\grave {Q}}$ is the mean of the modelled flows. The three statistical measurements were the one used in this study to evaluate the performance of the developed ANN model.

## Results and discussion

The result of this study is presented in three categories, namely; the descriptive statistics, the water quality test result and the ANN model and the model evaluation performance, respectively.

*The descriptive statistics result* is presented in Tables 1, 2, 3, 4. This describes the basic features of the data in this study. They provide simple summaries about the sample and the measures such as the mean, median, maximum, minimum and standard deviation, respectively.

**Table 1:** Descriptive statistics of the analyzed water quality at point 1.

Parameters	pH	TDS (mg/l)	EC (dS/m)	Na^+^ (mg/l)
Mean	6.34	2458.19	4.24	39.13
Median	6.36	2439.50	4.01	37.00
Max	6.48	2742.00	5.82	64.50
Min	6.09	2199.00	3.18	24.50
SD	0.08	127.36	0.83	9.94

**Table 2:** Descriptive statistics of the analyzed water quality at point 2.

Parameters	pH	TDS (mg/l)	EC (dS/m)	Na^+^ (mg/l)
Mean	6.29	2265.40	3.54	41.18
Median	6.31	2241.00	3.25	35.44
Max	6.49	2523.00	4.85	77.31
Min	6.01	2044.00	3.01	21.21
SD	0.13	127.07	0.49	14.06

**Table 3:** Descriptive statistics of the analyzed water quality at point 3.

Parameters	pH	TDS (mg/l)	EC (dS/m)	Na^+^ (mg/l)
Mean	6.33	2132.88	3.35	48.01
Median	6.37	2104.00	3.25	40.95
Max	6.48	2404.00	3.84	88.45
Min	6.00	1883.00	3.01	29.24
SD	0.10	114.47	0.23	14.14

**Table 4:** Descriptive statistics of the analyzed water quality at point 4.

Parameters	pH	TDS (mg/l)	EC (dS/m)	Na^+^ (mg/l)
Mean	6.34	1956.21	2.89	51.06
Median	6.39	2010.00	3.14	50.25
Max	6.64	2286.00	2.21	71.24
Min	6.09	1367.00	2.01	40.24
SD	0.16	213.04	31.49	8.16

The descriptive statistics in Tables 1,2, 3, 4 shows that the mean values of the data set ranges from 6.29 to 6.34, 1956.21 to 2458.19, 3.35 to 7.39 and 39.13 to 51.06 for Ph, TDS (mg/l), EC (dS/m) and Na (mg/l), respectively. The median values of the data set ranges from 6.31 to 6.39, 2010.00 to 2439.50, 3.14 to 4.24 and 39.13 to 51.06 for pH, TDS (mg/l), EC (dS/m) and Na (mg/l), respectively. The Maximum values data set ranges from 6.48 to 6.64, 2286.00 to 2742.00, 2.21 to 5.82, and 64.50 to 88.45 for Ph, TDS (mg/l), EC (dS/m) and Na (mg/l), respectively. The minimum values dataset ranges from 6.00 to 6.09, 1367.00 to 2199.00, 2.01 to 3.18, and 21.21 to 40.24 for Ph, TDS (mg/l), EC (dS/m) and Na (mg/l), respectively. The standard deviation values ranges from 0.08 to 0.16, 114.47 to 213.04, 0.23 to 31.49 and 14.06 to 8.16 for Ph, TDS (mg/l), EC (dS/m) and Na (mg/l), respectively. The low values of standard deviation recorded in this study shows that data set were very close to the mean of the dataset.

The water quality analysis test result indicates the level of concentrations of the TDS (mg/l), EC (dS/m) and Na (mg/l) in the Ele river in Nnewi, Anambra State Nigeria. The FAO standard for irrigation water quality for TDS, EC and Na are 0–2000, 0–3 and 0–40, respectively. The water quality results show that the pH values which ranges from 6.01 to 6.87 were within the FAO standard in all the points for both rainy and dry seasons, whereas the TDS (mg/l), EC (dS/m) and Na (mg/l) parametric values range from 2001 to 2506, 3.01 to 5.76, and 40.42 to 73.45 respectively, were above the FAO standard from point 1 to point 3 and falls within the FAO standard at point 4 with values ranging from 1003 to 1994, 2.01 to 2.78 and 31.24 to 39.44, respectively. However, during the dry season, the TDS, EC, and Na values range from 2002 to 2742, 3.04 to 5.82 and 40.14 to 88.45 respectively, were all above the FAO standard. Anthropogenic pollution emitted into water bodies has recently been identified as a significant source of pollutants that need immediate action in order to avoid serious environmental effects^11^.

The results equally revealed that the concentrations decrease along the sampling points going downstream. It is noteworthy that irrigation water with a pH outside the normal range may cause a nutritional imbalance or may contain a toxic ion which is harmful to crops^19^. The high concentrations of TDS as observed in this study are likely to increase the salinity of the river water, change the taste of the water, and as well decrease the dissolved oxygen level of the surface water making it difficult for the survival of plants and aquatic organisms^7^.

Moreover, these anions and cations which increase the electric conductivity in water affect irrigation adversely since salts settle at crop root zones making it difficult for infiltration, absorption of moisture and nutrients necessary for crop production.

*The ANN model and forecast* for the water quality parameters are shown from Figs. 4, 5, 6, 7, 8, 9, 10, 11, 12, 13, 14, 15, 16, 17, 18, 19. Considering the water quality permissible range, River quality modeling and forecast shows different variations seasonally such that the pollution level during dry season was higher than the rainy season.

**Figure 4:**
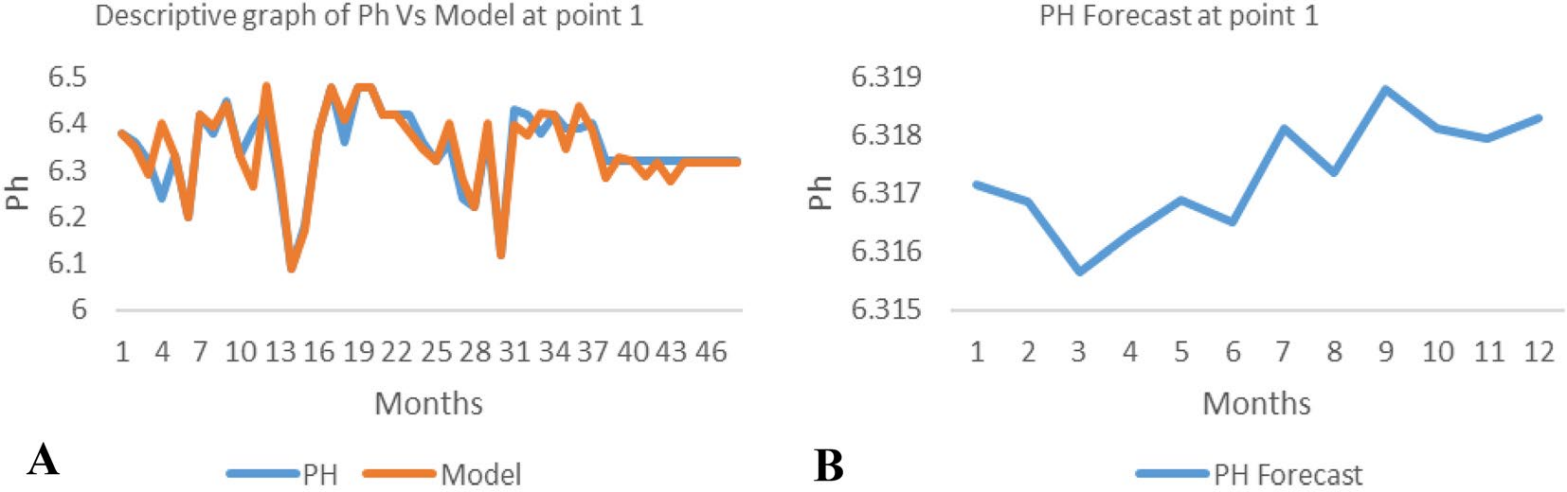
(**A** and **B**): pH model and forecast graph at point 1.

**Figure 5:**
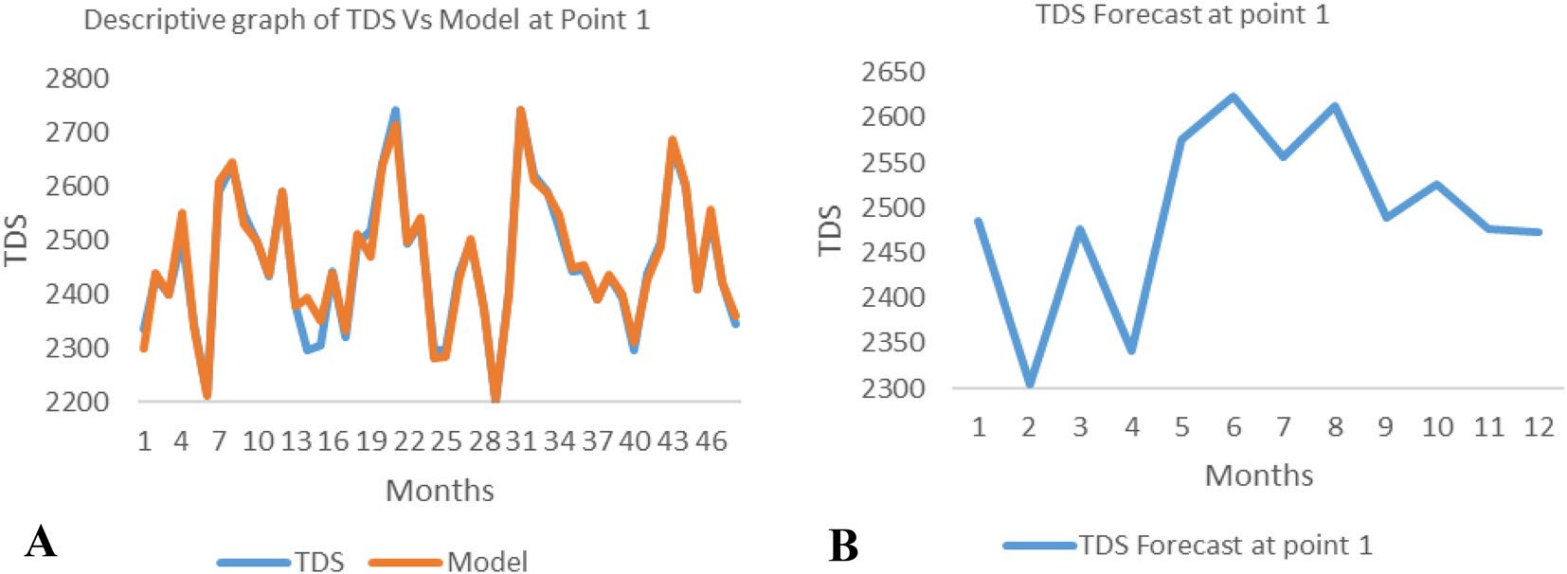
(**A** and **B**): TDS model and forecast graph at point 1.

**Figure 6:**
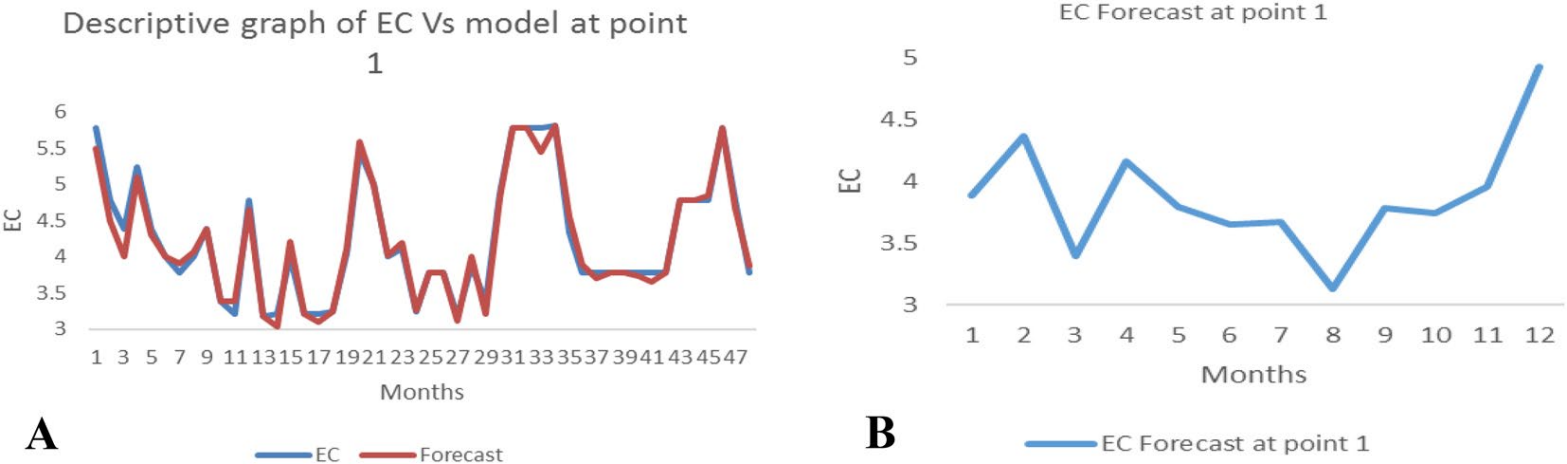
(**A** and **B**): EC model and forecast graph at point 1.

**Figure 7:**
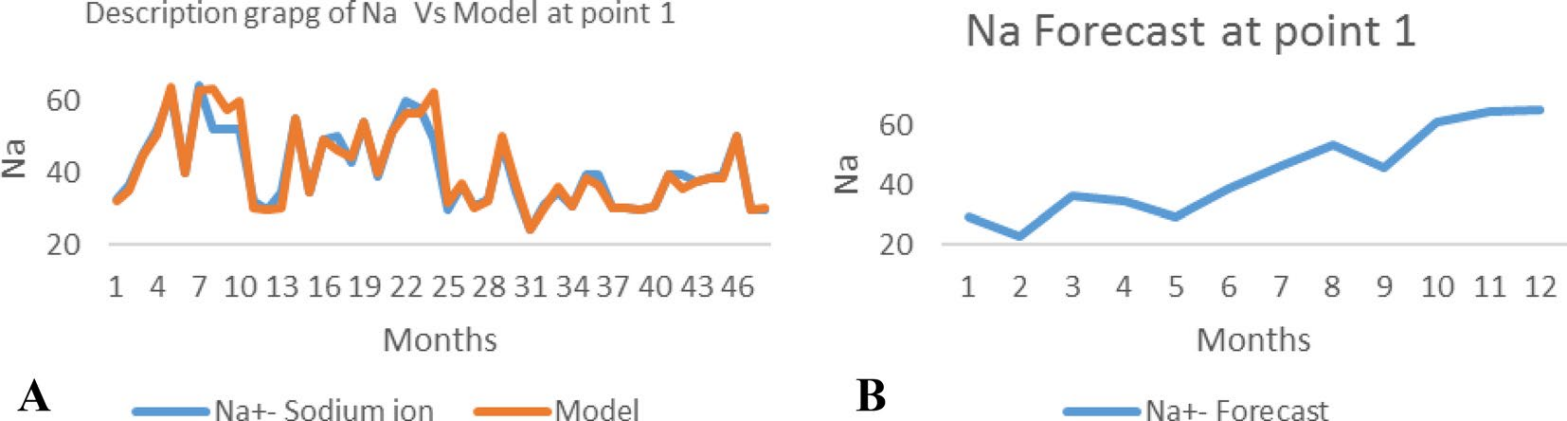
(**A** and **B**): Na model and Forecast graph at point 1.

**Figure 8:**
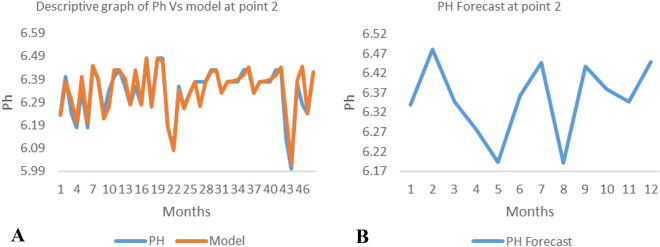
(**A** and **B**): Ph model and Forecast graph at point 2.

**Figure 9:**
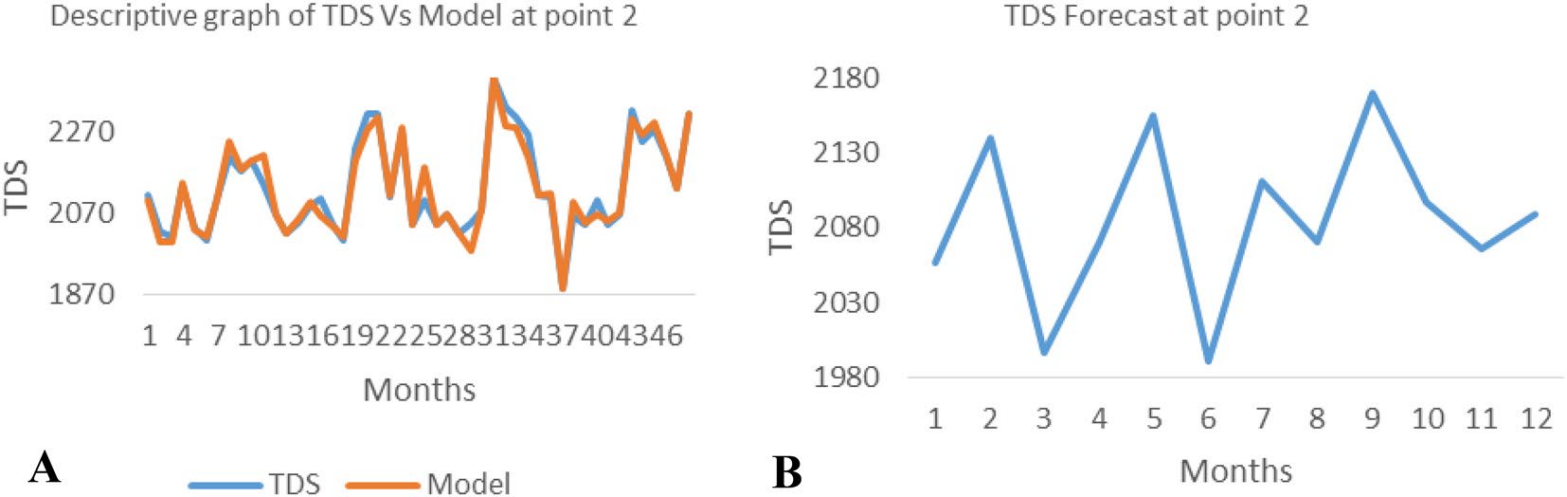
(**A** and **B**): TDS model and Forecast graph at point 2.

**Figure 10:**
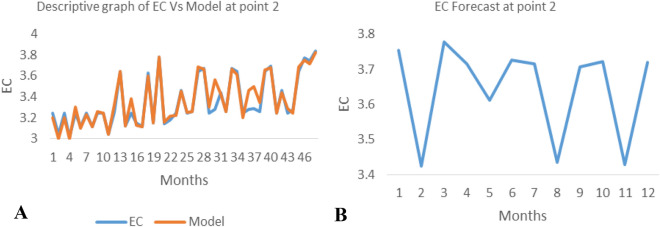
(**A** and **B**): EC model and Forecast graph at point 2.

**Figure 11:**
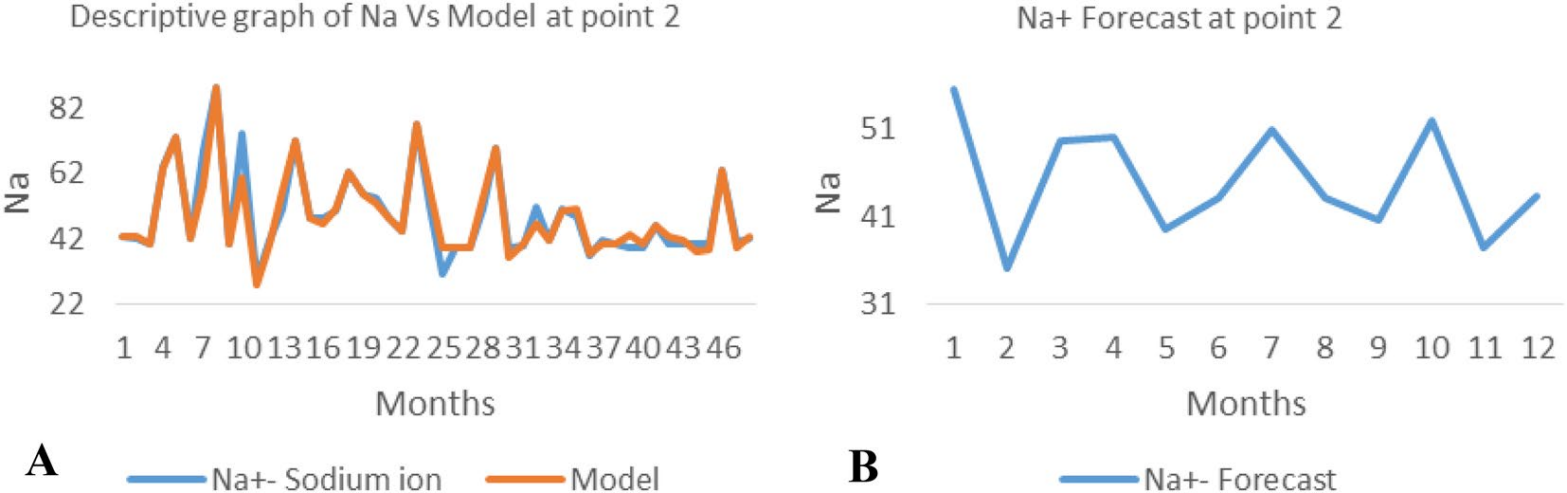
(**A** and **B**): Na model and Forecast graph at point 2.

**Figure 12:**
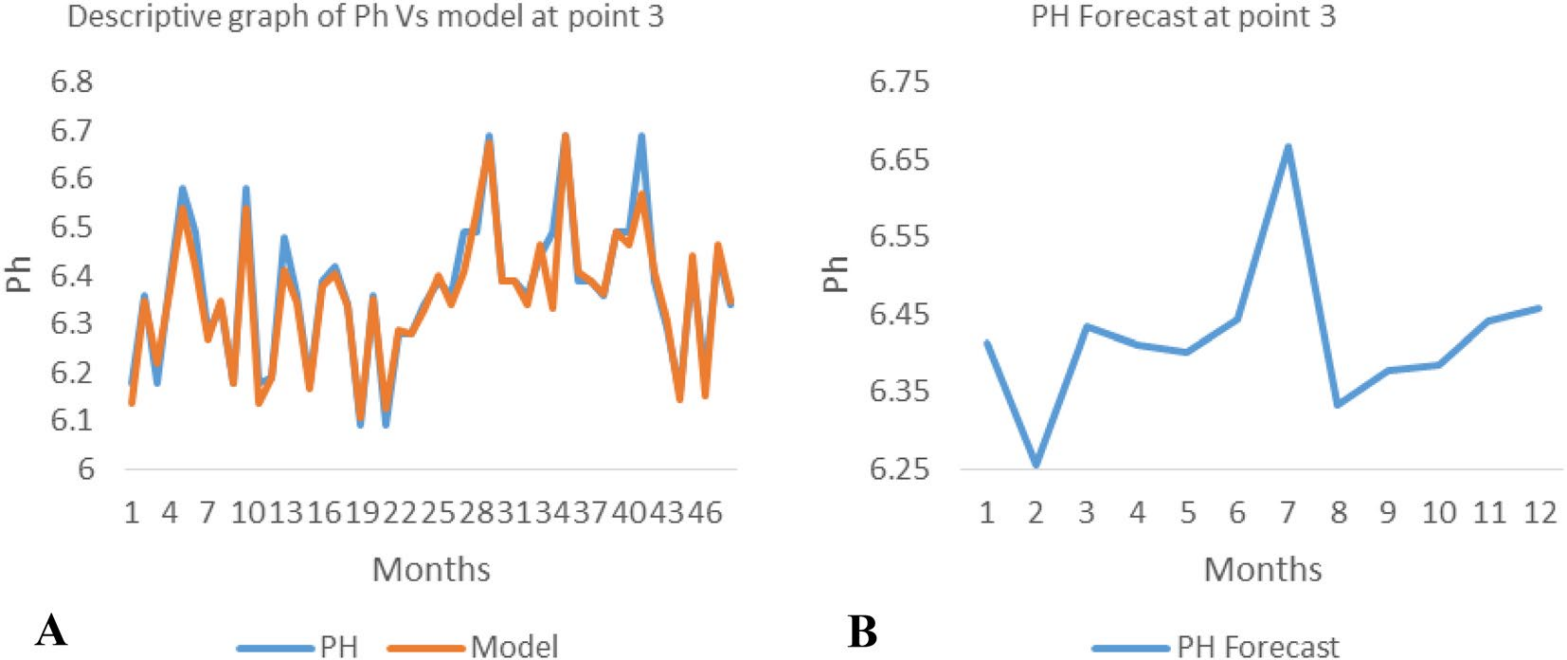
(**A** and **B**): Ph model and Forecast graph at point 3.

**Figure 13:**
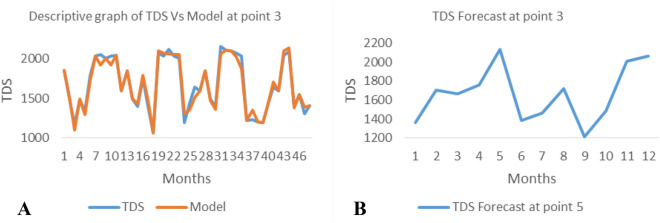
(**A** and **B**): TDS model and Forecast graph at point 3.

**Figure 14:**
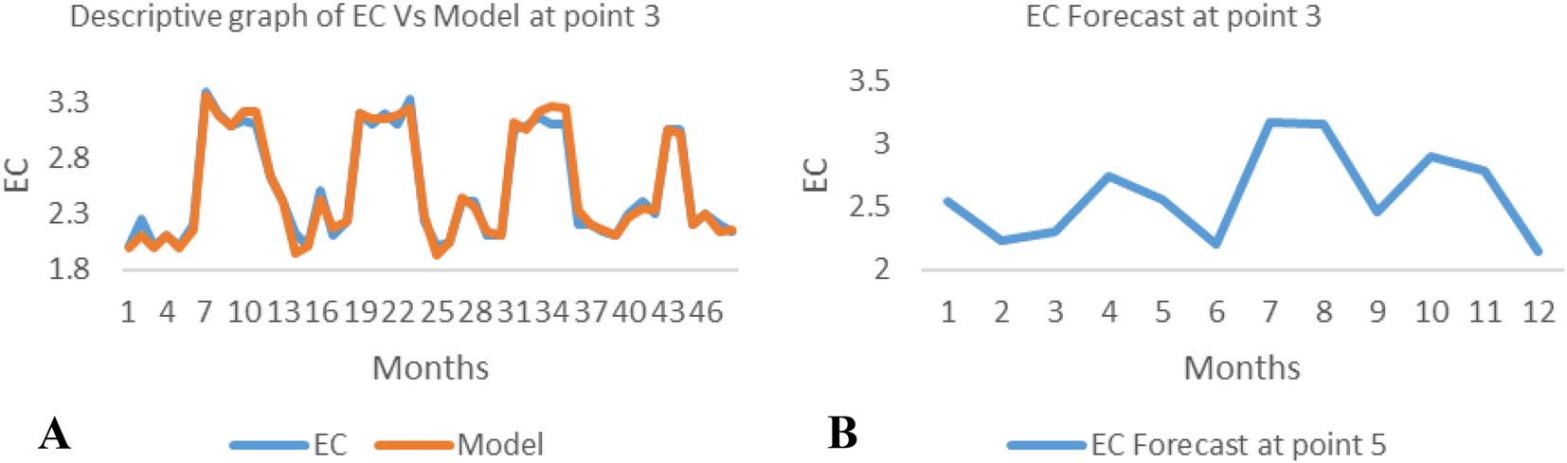
(**A** and **B**): EC model and Forecast graph at point 3.

**Figure 15:**
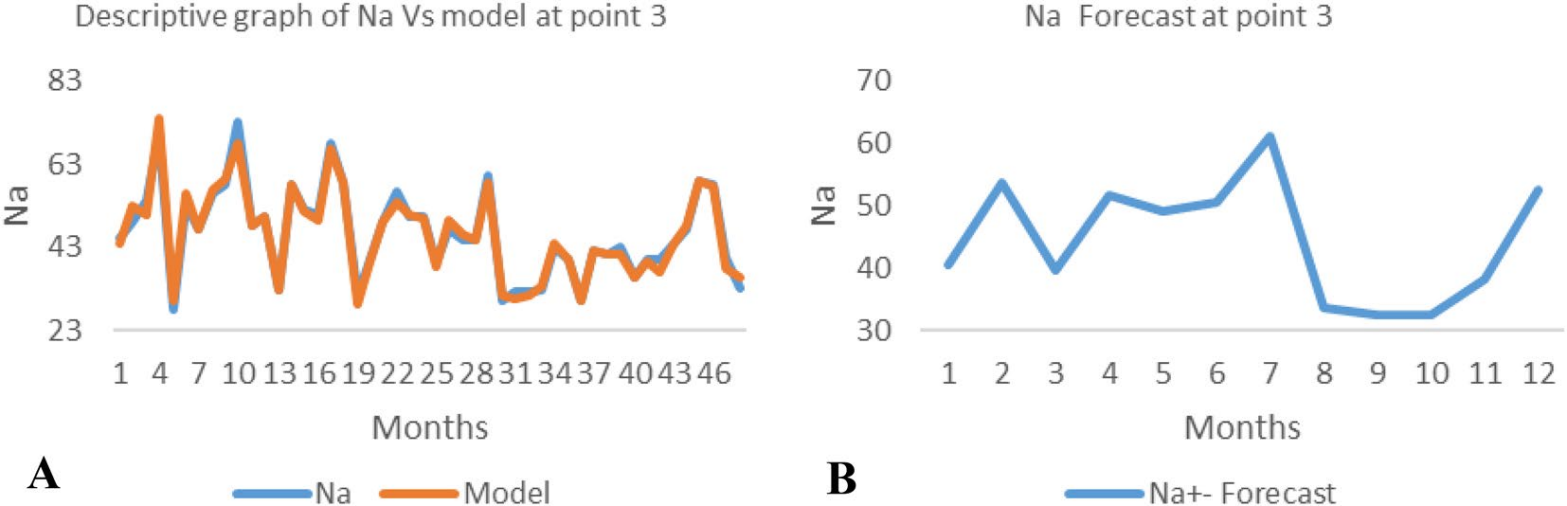
(**A** and **B**): Na model and Forecast graph at point 3.

**Figure 16:**
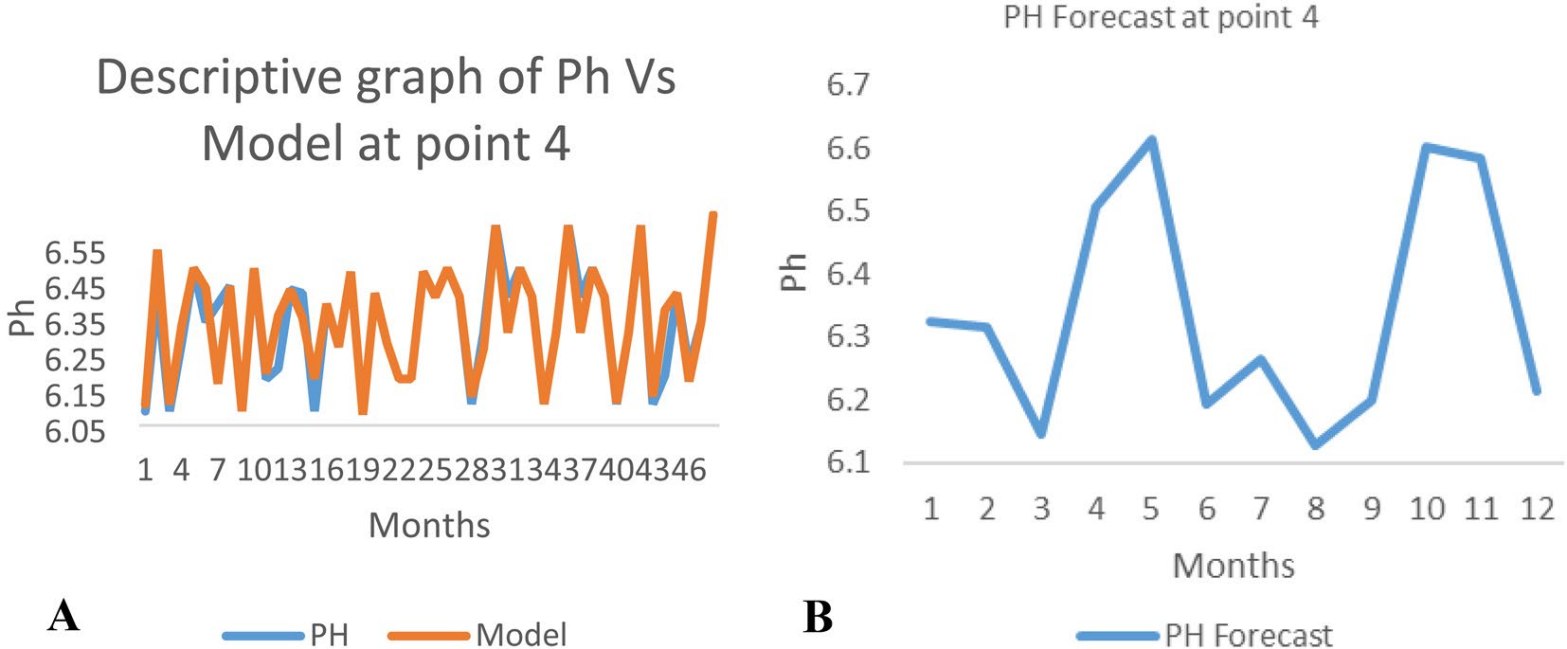
(**A** and **B**): pH model and Forecast graph at point 4.

**Figure 17:**
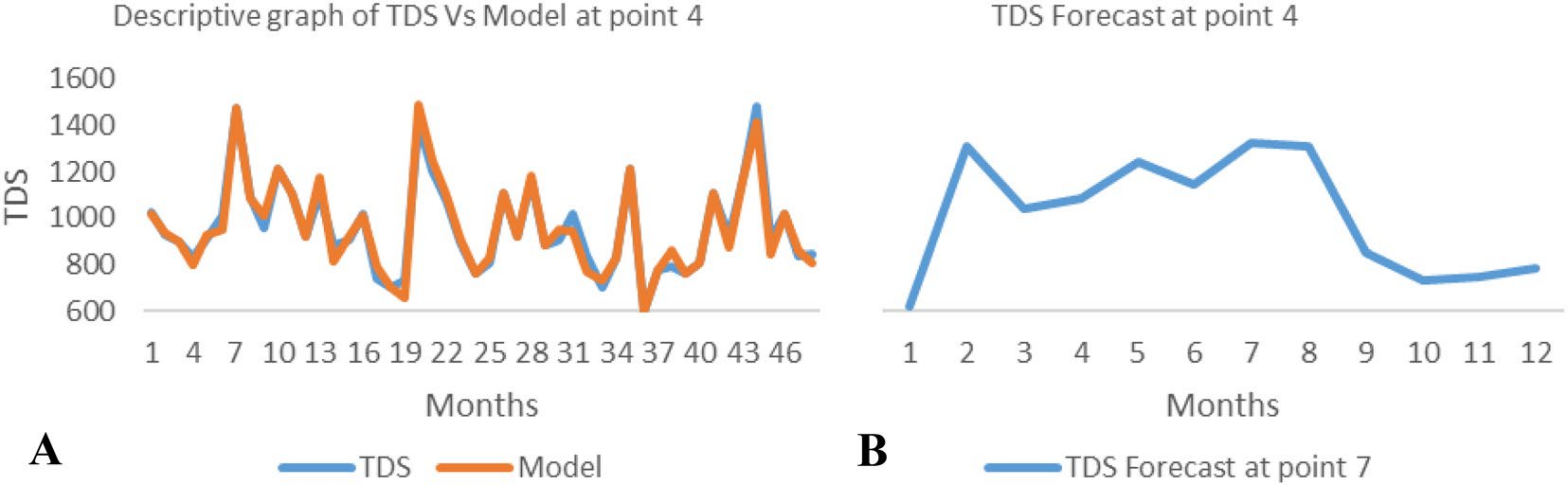
(**A** and **B**): TDS model and Forecast graph at point.

**Figure 18:**
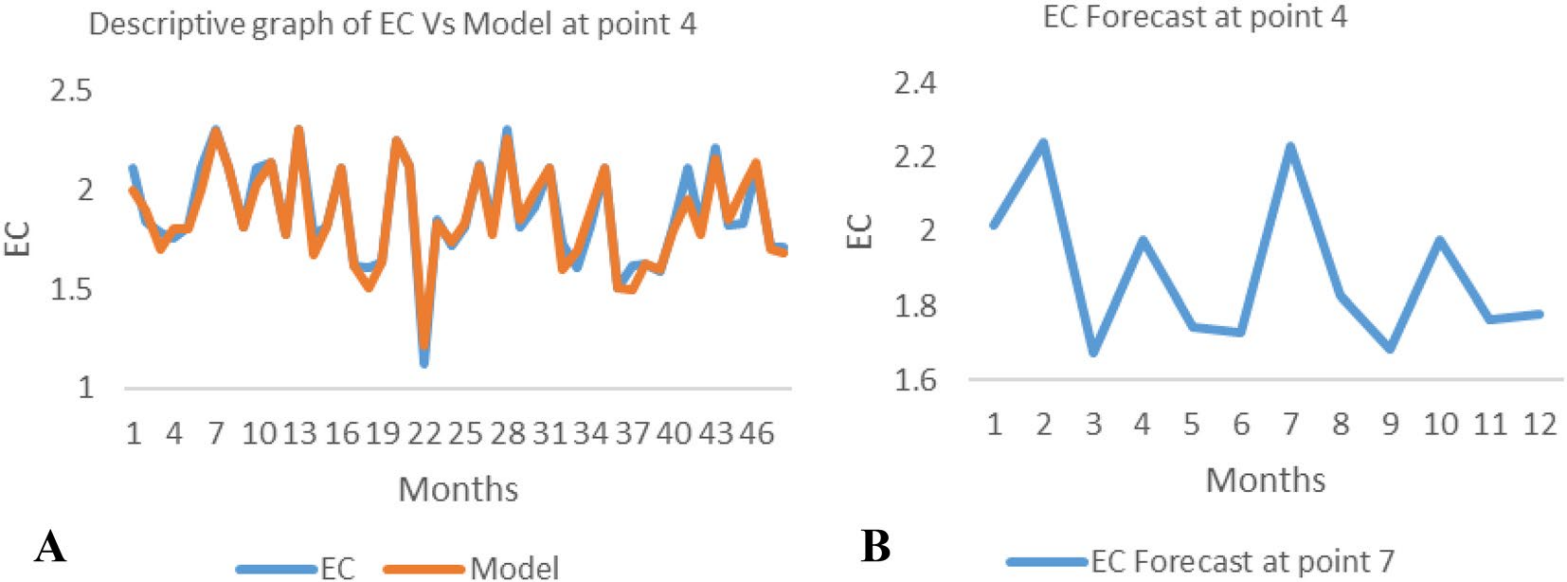
(**A** and **B**): EC model and Forecast graph at point 4.

**Figure 19:**
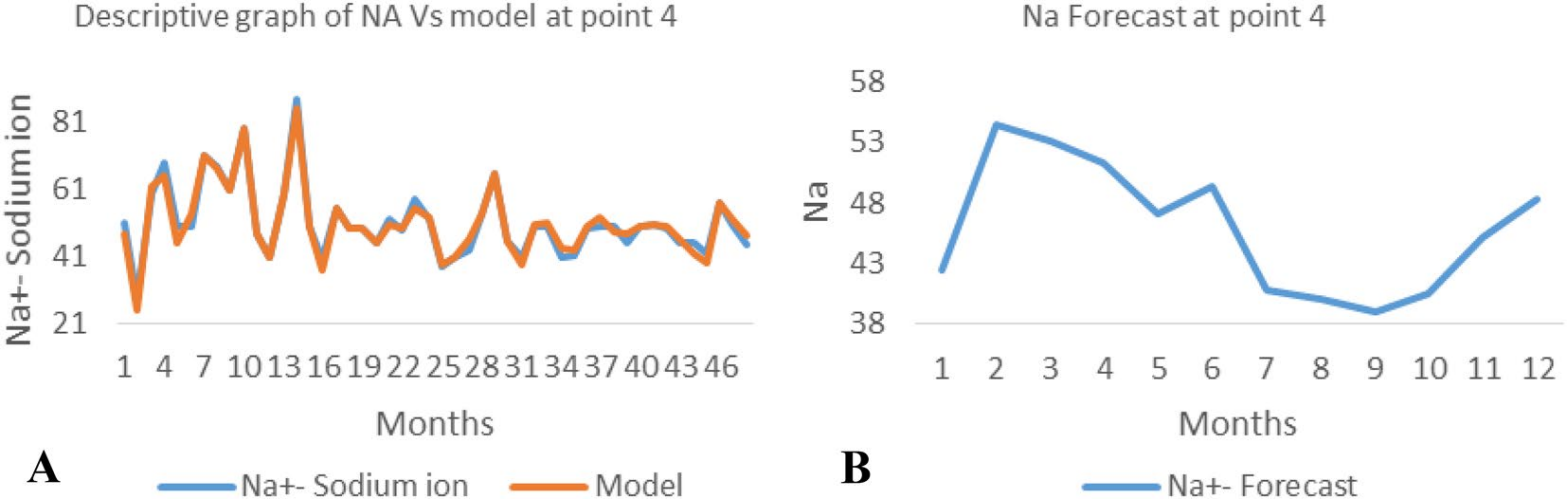
(**A** and **B**): Na model and Forecast graph at point 4.

Generally, the artificial neural network model the actual data set very well. At various sampling points, the developed ANN models descriptively show insignificant values in deviation for the actual data set. There were continues variations in the developed models and forecasts over time. 

*The feed-forward Multilayer Neural Network (FFMNN) Model Performance Evaluation Results* are shown in Table 5. The model performance evaluation was carried out based on the developed ANN model training, Testing and forecast, respectively. The model performance evaluation was carried out using the coefficient of multiple determination R^2^ and Root Mean Squared Error (RMSE).

**Table 5:** Statistical measurement of the trained, test and forecast model.

Parameters	Stat. measurement	P1	P2	P3	P4
Ph	RSQUAD TRAIN	0.981	0.983	0.982	0.990
	RSQUAD TEST	0.956	0.955	0.959	0.955
	RSQUAD FORECAST	0.962529	0.965922	0.959714	0.948418
	RMSE	0.048519	0.022088	0.026023	0.043385
TDS	RSQUAD TRAIN	0.981	0.985	0.982	0.981
	RSQUAD TEST	0.959	0.970	0.964	0.954
	RSQUAD FORECAST	0.947682	0.965259	0.949851	0.945909
	RMSE	0.043497	0.046244	0.011687	0.055819
EC	RSQUAD TRAIN	0.989	0.982	0.984	0.982
	RSQUAD TEST	0.967	0.959	0.951	0.954
	RSQUAD FORECAST	0.952864	0.95328	0.946784	0.947022
	RMSE	0.015462	0.017259	0.028919	0.080258
Na	RSQUAD TRAIN	0.986	0.984	0.988	0.985
	RSQUAD TEST	0.966	0.953	0.968	0.956
	RSQUAD FORECAST	0.961157	0.958157	0.950361	0.94929
	RMSE	0.031175	0.067912	0.023904	0.013521

*TDS* total dissolved solids; *EC* Electrical conductivity; *Na* Sodium, *mg/l* milligrams per litre; *dS/m* Deci-siemens per metre.

The R^2^ values were generally observed to have varied in the second decimal place for the training, testing and forecast model, respectively.

The training performance evaluation shows that R^2^ values ranges from 0.981 to 0.990, 0.981 to 0.988, 0.981 to 0.989 and 0981 to 0.989, for pH, TDS, EC, and Na, respectively. The training results shows that the pH model have the best performance followed by EC, and Na.

Also, the testing performance shows that the R^2^ value ranges from 0.952 to 0.967, 0.953 to 0.970, 0.951 to 0.967 and 0.953 to 0.968, for pH, TDS, EC and Na, respectively. However, the testing performance evaluation shows that TDS had the best performance. The forecast performance evaluation shows that the R^2^ values ranges from 0.945 to 0.968, 0.946 to 0.968, 0.944 to 0.967 and 0.949 to 0.965 for pH, TDS, EC and Na respectively. It was however discovered that the TDS made best forecast followed by the pH. The water quality forecast performance was further evaluated using the Root Mean Squared Error (RMSE) which ranges from 0.022 to 0.088, 0.012 to 0.087, 0.015 to 0.085and 0.014 to 0.084 for pH, TDS, EC and Na, respectively. The ANN model performed very well as their coefficient of multiple determinations R^2^ were very close 1, which is in agreement with the study of Awu et al. (2017) and Abrahart et al., (2005). On comparing the performance of the training model to the testing model and forecast, it shows that the training set performed better than the testing set followed by the forecast as its coefficient of multiple determinations, R^2^, was much closer to 1.

## Conclusion

Considering the FAO Irrigation water quality permissible standards, the River water quality analyses, modeling and prediction were evaluated for Ele River Nnewi, Anambra State. Results obtained showed that TDS, EC and Na were above the FAO permissible standard for irrigation during dry seasons while the pH was normal all through the season. The R^2^ values obtained from the water quality index and prediction were very close to 1 indicating a good model and prediction. Since this work is limited to irrigation water quality assessment, I recommend that future works on the water quality of Ele River can also determine the drinking and domestic water quality assessment using Artificial Neural Network ANN.

## Data availability statement

Some data through which the mean, median, max, min and standard deviation were derived are available from the corresponding author upon reasonable request. Also scatters plots through which models and forecasts were derived which support the findings of this study are available from the corresponding author.

## Abbreviations

EC: Electric conductivity
FAO: Food and agricultural organization
FFMLP: Feed-forward multilayer perceptron
RMSE: Root mean squared equation
TDS: Total dissolved solid
